# THE IMPACT OF INTERGENERATIONAL SUPPORT ON DEPRESSIVE SYMPTOMS AMONG OLDER MEXICAN AMERICANS: DOES GENDER MATTER?

**DOI:** 10.1093/geroni/igz038.3013

**Published:** 2019-11-08

**Authors:** Yaolin Pei, Zhen Cong, Bei Wu

**Affiliations:** 1 New York University, New York, New York, United States; 2 The University of Texas at Arlington, Arlington, Texas, United States; 3 NYU Rory Meyers College of Nursing, New York, New York, United States

## Abstract

The study examined gender differences in the impact of living alone and intergenerational support on depressive symptoms among Mexican American older adults. The sample included 335 parent-adult child dyads which were nested within 92 Mexican American respondents in a city in West Texas. Each respondent reported their specific relationships with each child. The results from clustered regression showed that men provided and received less intergenerational support than women, but their depressive symptoms were more susceptible to living alone and different types of intergenerational support. Factors such as living alone, and receiving instrumental support were related to higher levels of depressive symptoms among Mexican American older men than among in their female counterparts, whereas emotional closeness with children was associated with lower level of depressive symptoms in men than in women. The findings can be used to develop and target a gender-specific approach for depression interventions among older Mexican Americans.

